# Supplementary material for: Charge density wave induced nodal lines in LaTe3
Source: Nat Commun. 2023 Jun 19;14:3628. doi: 10.1038/s41467-023-39271-1 (PMC10279677; doi:10.1038/s41467-023-39271-1)
Supplement: Supplementary file 1 — Supplementary Information [file 41467_2023_39271_MOESM1_ESM.pdf]

Supplementary information for "Charge density wave induced nodal lines in $LaTe_3$"

Shuvam Sarkar[1], Joydipto Bhattacharya[2,3], Pampa Sadhukhan[1], Davide Curcio[4], Rajeev Dutt[2,3], Vipin Kumar Singh[1], Marco Bianchi[4], Arnab Pariari[5], Shubhankar Roy[6], Prabhat Mandal[5], Tanmoy Das[7], Philip Hofmann[4], Aparna Chakrabarti[2,3], Sudipta Roy Barman[1†]

[1]*UGC-DAE Consortium for Scientific Research, Khandwa Road, Indore 452001, Madhya Pradesh, India*

[2]*Theory and Simulations Laboratory, Raja Ramanna Centre for Advanced Technology, Indore 452013, Madhya Pradesh, India*

[3]*Homi Bhabha National Institute, Training School Complex, Anushakti Nagar, Mumbai 400094, Maharashtra, India*

[4]*Department of Physics and Astronomy, Interdisciplinary Nanoscience Center (iNANO), Aarhus University, 8000 Aarhus C, Denmark*

[5]*Saha Institute of Nuclear Physics, HBNI, 1/AF Bidhannagar, Kolkata 700 064, India*

[6]*Vidyasagar Metropolitan College, 39, Sankar Ghosh Lane, Kolkata 700006, India*

[7]*Department of Physics, Indian Institute of Science, Bangalore, 560012, India and*

[†]*barmansr@gmail.com*

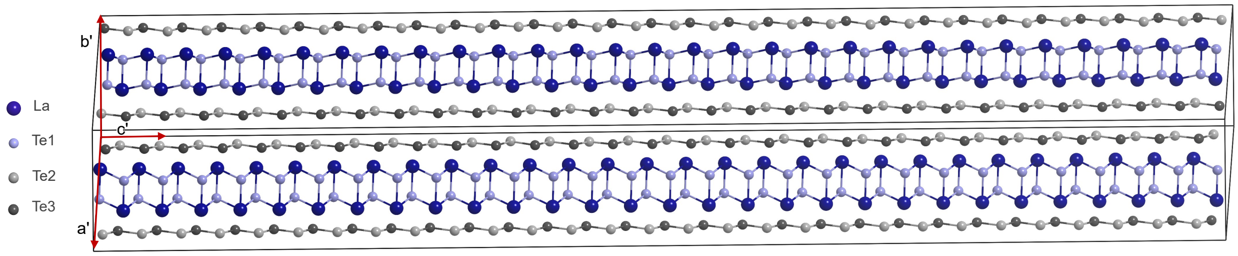

Supplementary Fig. 1. **The 29 fold (29f) structure of LaTe$_3$.** The primitive unit cell of the 29-fold (29f) structure (1×1×29) of LaTe$_3$ comprising of 232 atoms with $\mathbf{q}_{\mathrm{CDW}} = \frac{8}{29}\mathbf{c}^* = 0.2759\mathbf{c}^*$ viewed perpendicular to the $\mathbf{c}'$ direction. The lattice constants are $a' = b' = 13.256$ Å, $c' = 127.507$ Å with $\alpha' = \beta' = 90°$, and γ'=160.99°. It has the same space group ($C2cm$) as the 7f structure in Fig. 1a.

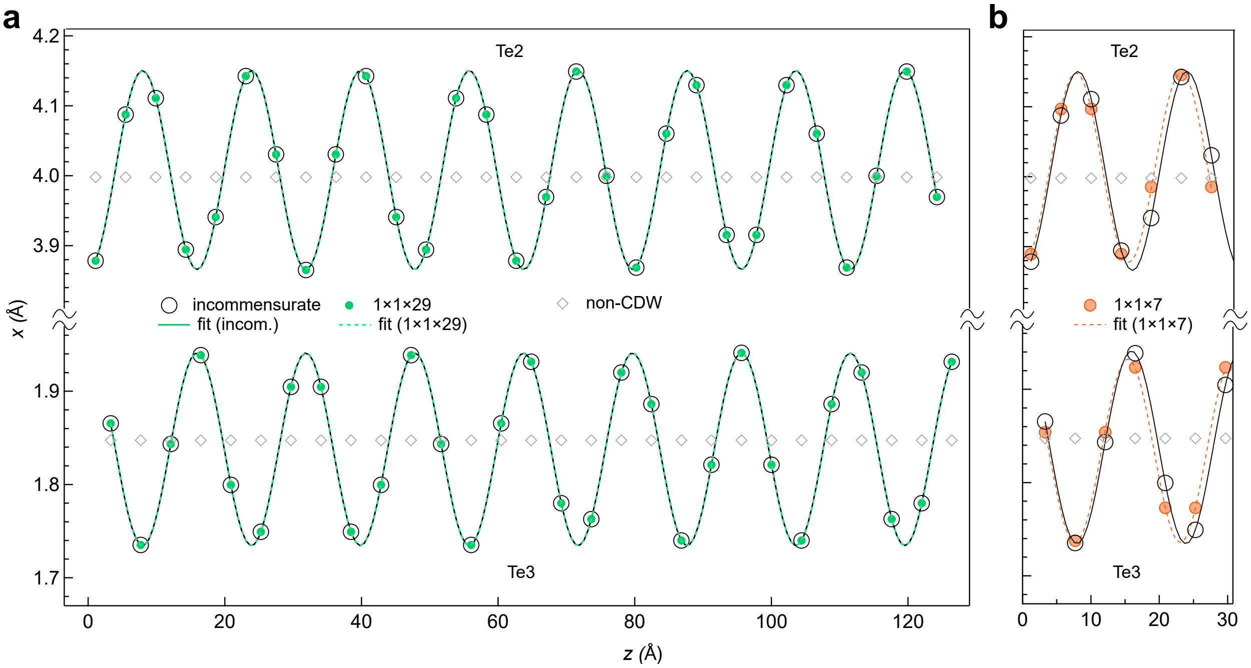

Supplementary Fig. 2. CDW modulation in the Te net of $LaTe_3$. Comparison of the Te (Te2 and Te3) atom positions in the x direction between the incommensurate structure obtained from x-ray crystallography [1] with **a** the 29-fold (29f) structure and **b** the 7-fold (7f) structure. The diamond markers denote the positions of undistorted Te atoms in the non-CDW state. $\mathbf{q}_{\mathrm{CDW}}$ determined from a sinusoidal fit for the x-ray crystallography structure is in agreement with the 29f structure ($0.2759\mathbf{c}^*$) within experimental accuracy. The atoms positions, the amplitude and the phase of the modulation wave are indistinguishable between the two. This is also true for the y and z directions, where the amplitude is reduced by order of magnitude compared to x direction (see Supplementary Fig. 3). On the other hand, for the 7f structure small differences are visible, as mentioned in the main text.

Supplementary Note 1:
Determination of $\mathbf{q}_{\mathrm{CDW}}$ from scanning tunneling microscopy and low energy electron diffraction

A scanning tunneling microscopy (STM) topography image in Supplementary Fig. 4a reveals the topmost Te layer, a zoomed image (white square) is shown in Fig. 1b of the main text. In Supplementary Fig. 4b, the Fourier transform of the STM image in panel **a** shows the CDW related satellite spots (blue circles) along $\mathbf{c}^*$. The intensity line profiles along AB and A′B′ in Supplementary Fig. 4c gives $\mathbf{q}_{\mathrm{CDW}} = 0.28 \pm 0.02\mathbf{c}^*$ that is related to the first

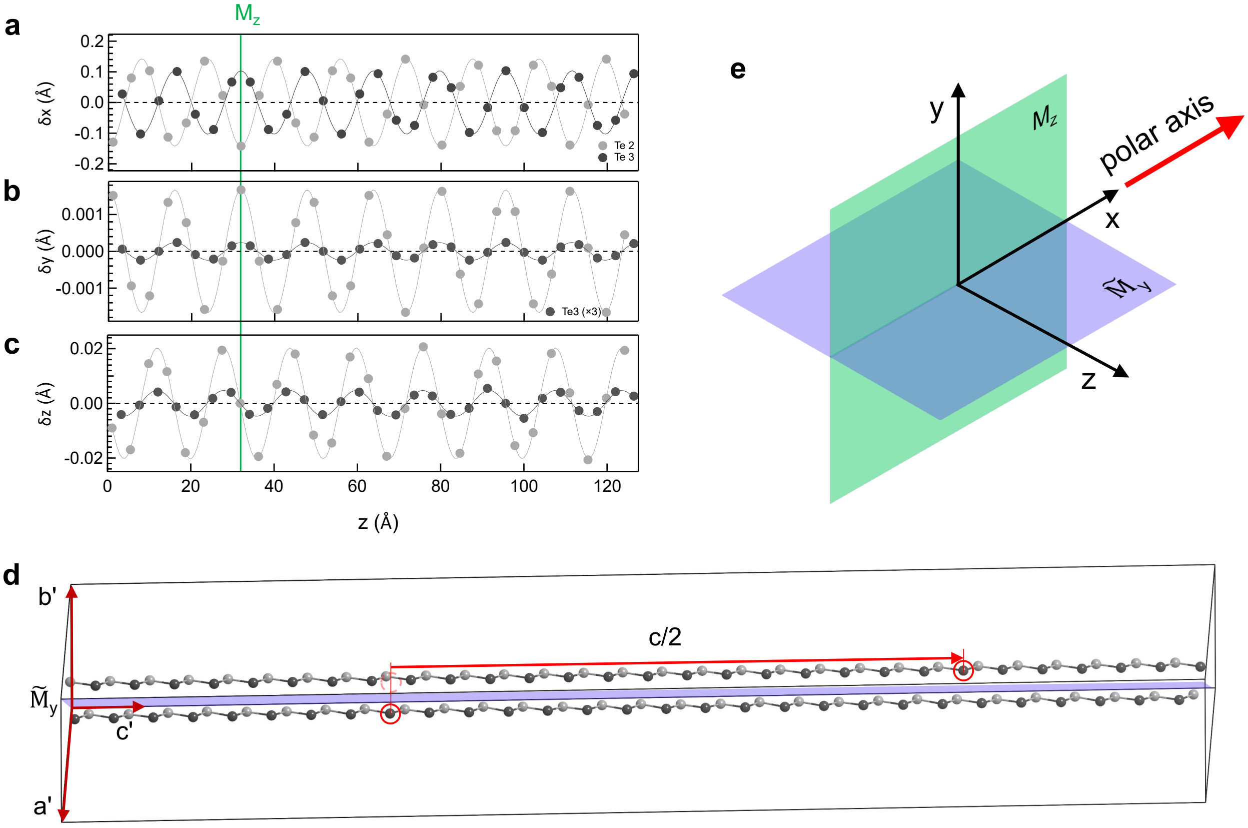

Supplementary Fig. 3. **Noncentrosymmetry and the polar axis of** $\mathbf{LaTe_3}$ **in the CDW state.** The distortion for the Te atoms (Te2 and Te3) that host the CDW along **a** x (δ_x), **b** y (δ_y), and **c** z (δ_z) axes. δ_x breaks the M_x mirror symmetry in the CDW state, the M_x mirror can be visualized to be perpendicular to the plane of the paper along the dashed line in panel **a**. However, these distortions preserve the M_z mirror symmetry as shown by the vertical green line in panels **a, b,** and **c**. **d** The glide symmetry (i.e., $\tilde{M}_y$ reflection and c/2 translation) is also preserved in the CDW structure. For clarity, only the Te2 bilayer is shown. **e** The polar axis that has to be parallel to both the M_z mirror in $y-x$ plane and $\tilde{M}_y$ glide mirror in $x-z$ plane [2] occurs along the x direction.

satellite peak.

Intensity profiles through CD and C′D′ (Supplementary Fig. 4d) of the low energy electron diffraction (LEED) pattern in Fig. 1c provide the estimate of $\mathbf{q}_{\mathrm{CDW}}$ to be 0.28±0.01$\mathbf{c}^*$, in good agreement with ARPES (Supplementary Figs. 7a,b) and STM (Supplementary Fig. 4c). Furthermore, the closeness of $\mathbf{q}_{\mathrm{CDW}}$ between the surface sensitive measurements (ARPES, STM and LEED) and bulk-sensitive XRD [1] is consistent with the 2D nature of $LaTe_3$.

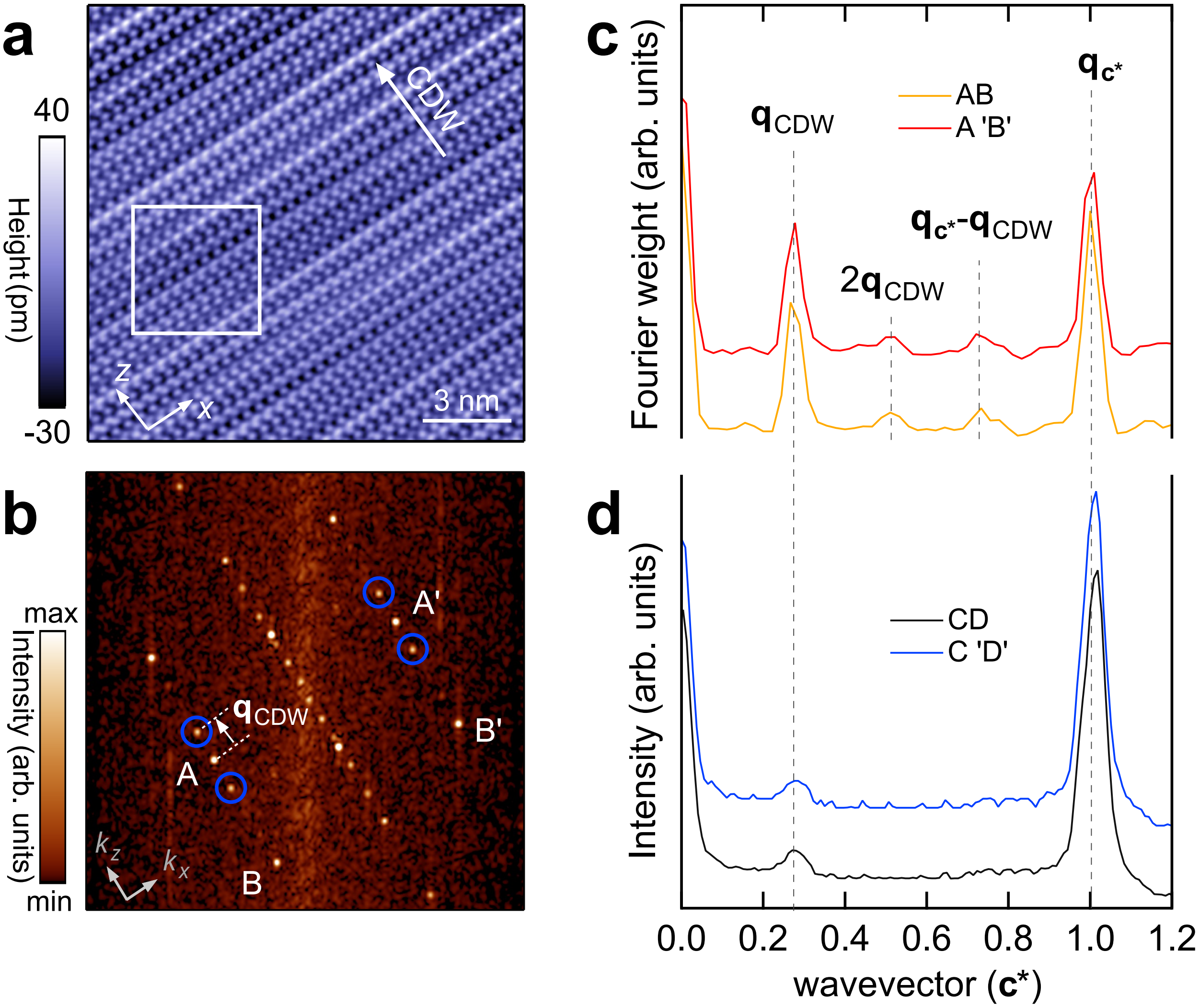

Supplementary Fig. 4. $\mathbf{q}_{\text{CDW}}$ **from STM and LEED. a** A large area (14.7×14.7 nm^2) scanning tunneling microscopy (STM) topography in the CDW state of $LaTe_3$ measured in the constant current mode with bias voltage of 0.2 V and a tunneling current of 0.4 nA. The zoomed image of the enclosed area inside the white box is shown in Fig. 1b in the main text. **b** Fast Fourier transform (FFT) of the STM image, where the CDW related satellite spots are highlighted by blue circles, $\mathbf{q}_{\text{CDW}}$ is the wave vector. The intensity line profiles from **c** the FFT of the STM image shown in panel **b** and **d** the low energy diffraction (LEED) pattern shown in Fig. 1**c** of the main text.

Supplementary Note 2:
Fermi surface of $LaTe_3$ in CDW state measured by ARPES

Shadow branches and determination of $\boldsymbol{q}_{\text{CDW}}$: The Fermi surface (FS) in Supplementary

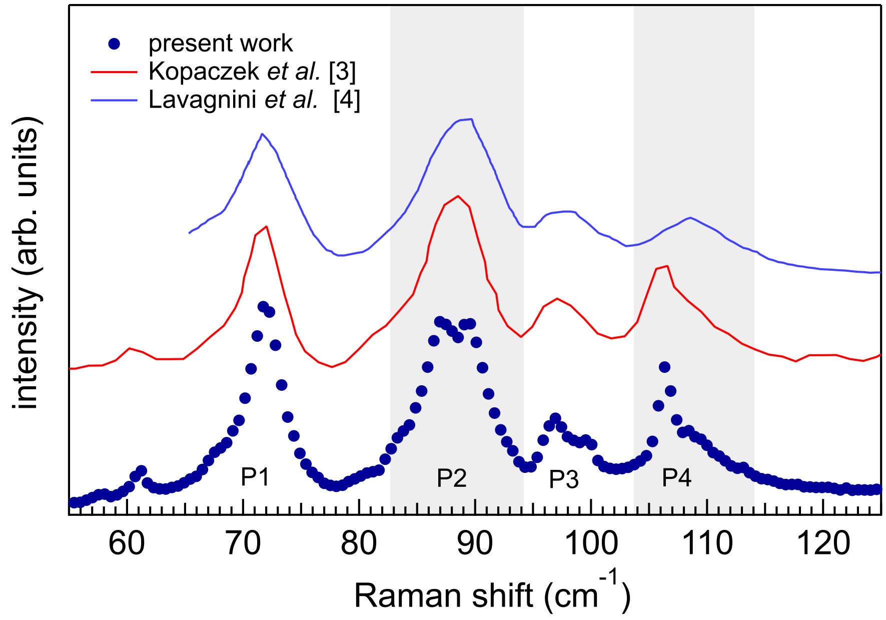

Supplementary Fig. 5. Raman spectrum of $LaTe_3$ in the CDW state. The Raman spectrum (dark blue circles) measured at 300 K shows four peaks indicated as P1, P2, P3 and P4. It is in good agreement with those reported by Kopaczek *et al.* [3] and Lavagnini *et al.* [4] that are shown staggered along the vertical axis by red and blue curves, respectively. The P2 and P4 peaks (highlighted by the gray rectangles) contain the B_1 symmetry mode which is an irreducible representation of C_{2v} point group and is a direct signature of noncentrosymmetry in $LaTe_3$ [4, 5].

Figs. 7a,b reveals the presence of weak FS branches highlighted by yellow arrows in the metallic part of the FS, besides the main branches. These are also observed in a larger area FS taken with 80 eV photon energy, see Supplementary Fig. 7d. The main branches are related to the main bands that are present in the non-CDW state, but the weak branches are absent in the calculated FS of the non-CDW state (Supplementary Fig. 7c, the different Fermi sheets are shown) indicating that these are related to the CDW state. These are nearly parallel to the sides of the γ pocket and connect with the ungapped corner of the α sheet. The ungapped portions of the outer β sheet (red arrows in Supplementary Figs. 7a,b) are also observed. If the β sheet is compared with the weak FS branch, it is clear that the latter is a replica of the former, but shifted by $\mathbf{q}_{\text{CDW}}$, as shown by a horizontal dashed black/blue arrow (#1). Moreover, a weak diamond shaped FS contour (one side denoted by blue arrow) is observed around the yellow dot, which is the replica of the γ pocket around the X point, again shifted by $\mathbf{q}_{\text{CDW}}$(black/blue dashed arrow #2). These additional branches

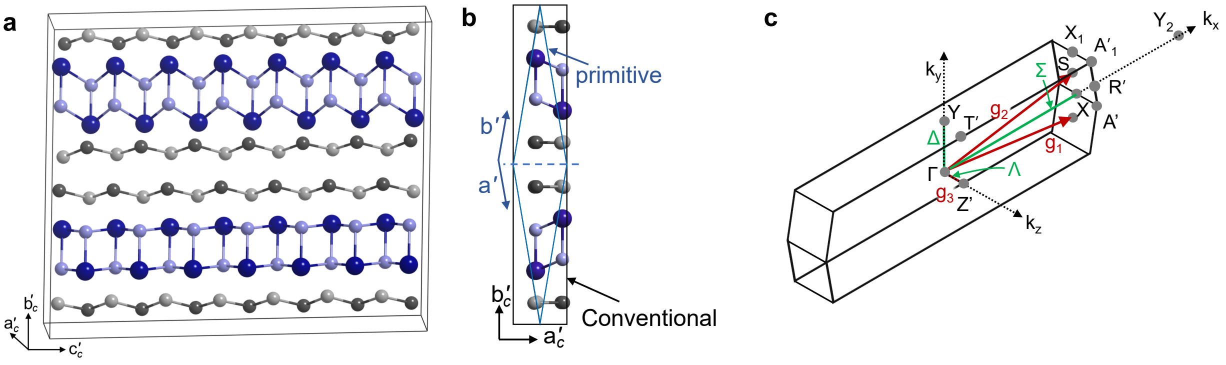

Supplementary Fig. 6. **The unit cell and Brillouin zone of $LaTe_3$ in the CDW state. a** The conventional i.e., non-primitive unit cell of the 7-fold modulated structure with 112 atoms and a'_c= 4.378, b'_c= 26.149, c'_c= 30.778 Å, α'_c= β'_c= γ'_c= 90°. A comparison with the lattice parameters of the *conventional* i.e., non-primitive unit cell of the non-CDW state with a_c= 4.378, b_c= 26.149, and c_c= 4.397 Å shows that c'_c is 7 times larger than c_c due to the 7-fold modulation. Also it is the convention in RTe_3 to take b_c as the vertical long axis, with Te bilayer in the a_c-c_c. **b** A comparison of the conventional and the primitive unit cell in the CDW state viewed along the c' axis. The blue dashed line represents the glide plane. **c** Brillouin zone (BZ) of the 7-fold structure of the CDW-state (ordering of the conventional lattice: $a'_c < b'_c$ [6]) showing the primitive reciprocal lattice vectors $\mathbf{g}_1$, $\mathbf{g}_2$ (both in k_x-k_y plane) and $\mathbf{g}_3$ (along k_z). The co-ordinates of the high symmetry points/lines in terms of the primitive reciprocal lattice vectors are as follows: Γ (0,0,0), X (ζ,ζ,0) where ζ= 0.257 = 0.25(1+a'^2/b'^2), Z' (0,0,0.5), Y (-0.5,0.5,0), A' (ζ,ζ,0.5), A'_1 (-ζ,1-ζ,0.5), T' (-0.5,0.5,0.5), S (0,0.5,0), R' (0,0.5,0.5), X_1 (-ζ,1-ζ,0) and lines Δ ($-u$,u,0), Λ (0,0,u), Σ (u,u,0), P (u,v,0), and M (u,u,v). Y_2 i.e., the Y point in the next BZ along Σ is given by (0.5,0.5,0).

(more prominent in the curvature plot in Supplementary Fig. 7b) are related to the shadow bands that arise due to the CDW periodicity [7]. The identification of the shadow branches of the FS allows a quantitative determination of $\mathbf{q}_{\text{CDW}}$ by taking cuts along k_z for different k_x. In this way, $\mathbf{q}_{\text{CDW}}$ is found to be $0.28\pm0.005\mathbf{c}^*$.

Bilayer splitting in the FS:

Bilayer splitting of the FS branches are observed in the β sheet (black dashed oval #1) and in the γ pocket around X (black dashed oval #2) in Supplementary Fig. 7a, which are

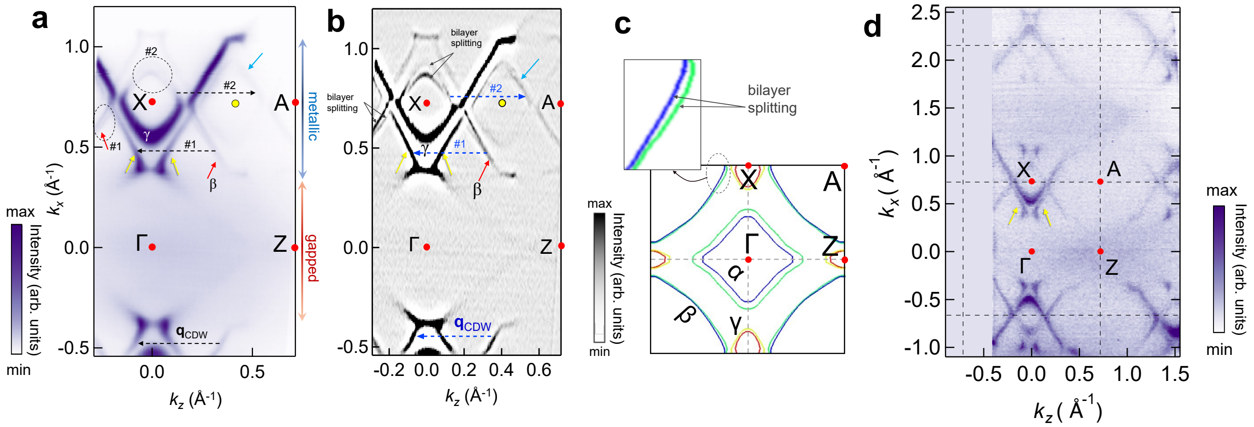

Supplementary Fig. 7. **Fermi surface from ARPES. a** The Fermi surface (FS) of $LaTe_3$ in the CDW state using photon energy (hν) of 24.4 eV. The gapped and the metallic parts of the FS are marked by red and blue double arrows on the right axis, respectively. The black dashed arrows of length $\mathbf{q}_{\rm CDW}$ connect some of the shadow branches with the main branches of the FS. The high symmetry points of the BZ (both CDW and non-CDW, see Fig. 1**d** of the main manuscript) are indicated. **b** A curvature plot of the FS shown in panel **a**, where the weak shadow FS pockets are visible. The blue dashed arrows indicate the $\mathbf{q}_{\rm CDW}$. **c** The calculated FS in the non-CDW state. The inset shows the bilayer splitting of the β sheet. **d** A large area FS plot measured using hν= 80 eV. The colorbars in panels **a**, **b**, and **d** show the intensity in arbitrary units (arb. units).

more prominent in the curvature plot (Supplementary Fig. 7b). The splitting varies with k, e.g., for the γ pocket it is maximum (0.05 Å^{-1}) along ΓX (Σ with coordinates (u,u,0) in terms of reciprocal lattice vectors, see Supplementary Fig. 6c) and becomes smaller for larger k_z. For the β sheet, it decreases for larger k_z. In contrast to the shadow branches, the calculated FS for the non-CDW state also shows such a splitting, as shown for the β sheet by a black dashed oval in Supplementary Fig. 7c and its inset. The bilayer splitting is also observed in the γ pocket, but is most prominent for the α sheet (green and blue curves).

Gapped part of the FS around ΓZ*:* The FS in Supplementary Fig. 7a shows a k_x= $\pm$0.4 Å^{-1} region around the ΓZ direction with no feature (vertical red double arrow on the right) in contrast to the metallic part (vertical cyan double arrow). This is because the FS here is gapped due to the CDW, in agreement with the ARPES band dispersion discussed in **Results** section of the main text (Fig. 4, Supplementary Figs. 7a,b and 11). In contrast,

the calculated FS of the non-CDW state in Supplementary Fig. 7c shows two diamond shaped sheets (α and β) around Γ and four smaller oval pockets (γ) around Z and X. The central part of the α and β sheets is not observed in the experimental FS as these are in the gapped region. However, the γ pocket around the X point is visible in the metallic region.

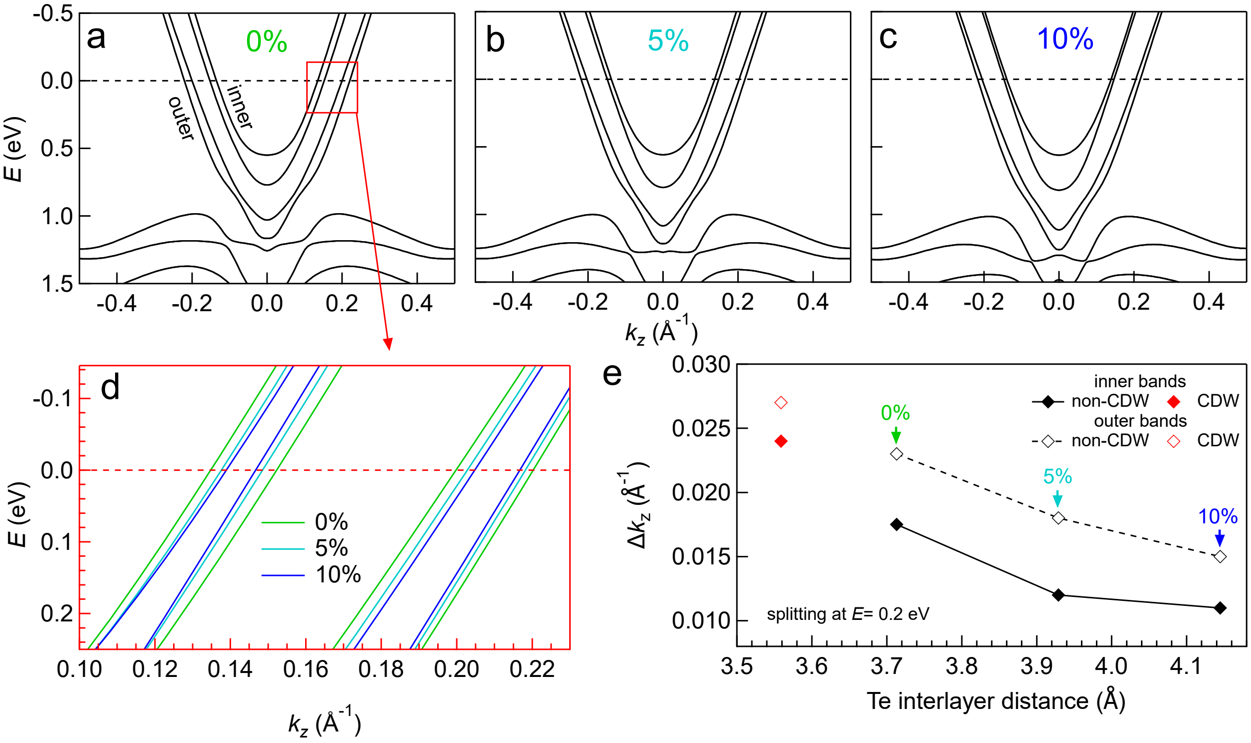

Supplementary Fig. 8. **Decrement of the bilayer splitting with increasing Te interlayer distance.** The band structure of the non-CDW state of $LaTe_3$ along k_z at k_x= 0.68 Å^{-1} for different Te interlayer distances: **a** 0% unchanged, **b** 5%, and **c** 10% increase. **d** The red box in panel **a** is zoomed to show the Δk_z. **e** Δk_z of the inner and outer bands at the E= 0.2 eV plotted as function of Te interlayer distance. As the Te interlayer distance increases, Δk_z for both the outer and inner bands of the non-CDW state decreases. Panel **e** also shows that Δk_z for the inner and outer bands of the CDW state (from Supplementary Fig. 15a) are close to the non-CDW values.

Supplementary Note 3: Bilayer splitting in the band structure

In order to probe the mechanism behind the bilayer-splitting, we have performed DFT

calculations for $LaTe_3$ in the non-CDW state by increasing the interlayer separation from 3.71 Å (0% increase) to 5% and 10% in the y direction keeping the rest of the interatomic distances - for example within the La-Te1 block- unchanged. Supplementary Fig. 8 shows that the bilayer splitting in Å^{-1}, determined at $E=$ 0.2 eV decreases as the interlayer separation increases. The fact that bilayer splitting exists in the non-CDW state shows that it is not related to the CDW. Furthermore, its decrease with increasing separation of the two layers establishes that the Te bilayer coupling is the mechanism behind the bilayer splitting.

To further substantiate the above propositions, in Supplementary Fig. 9 we have calculated the band structure of $LaTe_2$ that has one La-Te block and a single Te layer, the structure is shown in Supplementary Fig. 9a. This resembles the top half of the $LaTe_3$ structure i.e., above the blue plane in Supplementary Fig. 6b. Here, we find two bands around $k_z=0$ that are the inner and the outer main bands arising from the Te p states in the single layer. No splitting of these bands is observed since the Te bilayer is absent in this structure. A similar comparison has been performed by Laverock et. al. between $LuTe_3$ and $LuTe_2$ to show the absence of the bilayer splitting in the latter[8]. In spite of the absence of bilayer splitting in $LaTe_2$, it still hosts a CDW, as has been experimentally shown in literature [9, 10]. This again establishes that the CDW and bilayer splitting in rare-earth tellurides are independent phenomena.

The bilayer splitting in the CDW state is observed simply because the Te bilayer is present, as shown in Figs. 2h-k in the EBS and also in the corresponding folded band structure in Supplementary Figs. 15a-d. Quantitatively for $LaTe_3$, the bilayer splitting in the CDW state is not expected to be very different from the non-CDW state, because the CDW distortion is about 4% compared to the Te-Te distances. We show this similarity quantitatively in Supplementary Fig. 8e where the bilayer splitting from Supplementary Fig. 15a is plotted for comparison.

Supplementary Note 4: Bader charge analysis

The transfer of electrons from La to the Te2-Te3 net determines the band filling and this has been calculated using the Bader charge analysis [12, 13]. The results for the CDW and the non-CDW state are nearly similar, as shown in the Supplementary Table 1. We find that 0.5 electronic charge from a La atom is transferred to the two Te atoms in the net and

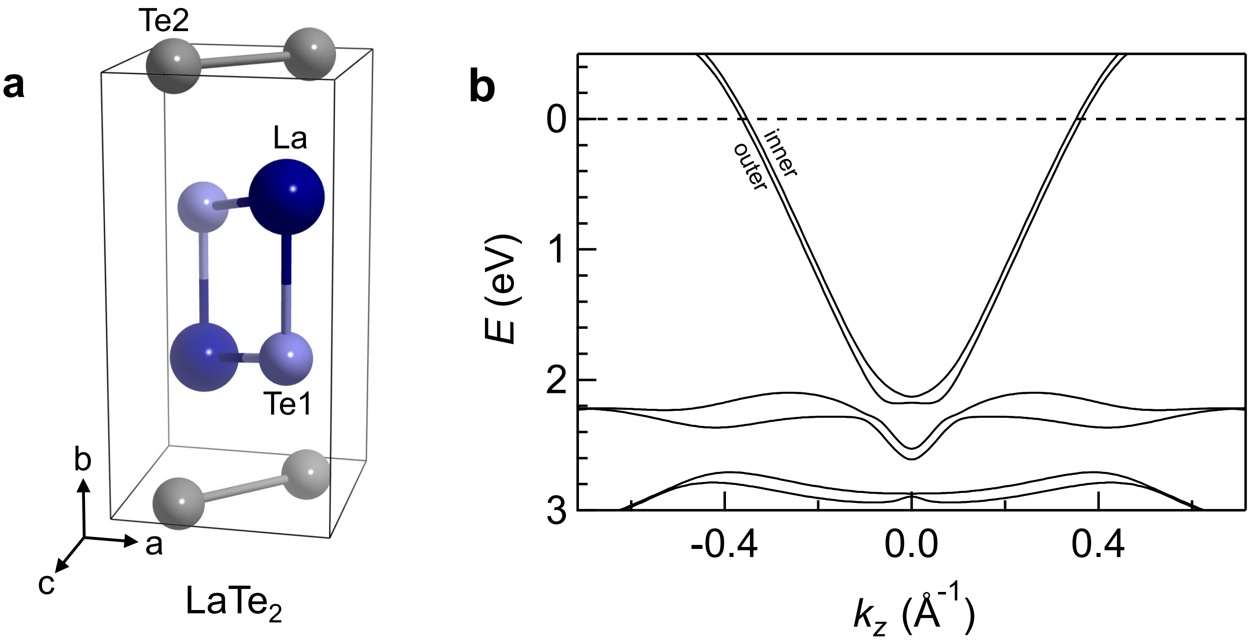

Supplementary Fig. 9. **Structure and band dispersion in $LaTe_2$. a** The crystal structure of $LaTe_2$ showing the single Te layer formed by Te2 and the La-Te1 block. **b** The band dispersion along the similar direction as in Supplementary Fig. 8.

thus the valency of both Te2 and Te3 is -0.25 (outer shell configurations of La and Te atoms are $5d^1 6s^2$ and $5s^2 5p^4$, respectively). One electron is transferred from La to Te1. Thus, the valency of La is +1.5, while that of Te1 is -1. The present DFT results thus do not support the assumption in an earlier tight binding calculation [14] that La has +3 valency. The two different valencies of Te is experimentally verified by the difference in the E of the Te2-Te3 and Te1 related peaks in the Te $4d$ core-level spectrum (Supplementary Fig. 10). The former is shifted to higher E (by 0.63 eV) as expected for lesser negative valency. Moreover, the integrated intensity of the Te2-Te3 peak being almost double of the Te1 peak justifies their identification. Two components in the Te $3d_{5/2}$ core level spectrum have also been reported from x-ray photoelectron spectroscopy [15].

Supplementary Note 5: Variation of the CDW gap with k_x and k_y

The CDW gap observed in the ARPES intensity plot towards the ΓZ [14, 16] is shown by a red double arrow in the first slice of Supplementary Fig. 11a (the same is shown in

Supplementary Table 1. Bader charge analysis of $LaTe_3$, where a positive sign of net charge transfer (Δ_Q) indicates that charge is donated, while a negative sign indicates that charge is accepted.

**No.**	**Atom**	**Δ_Q (e)**	
		**non-CDW**	**CDW**
1	La1	1.503	1.539
2	Te1	-0.996	-1.029
3	Te2	-0.252	-0.254
4	Te3	-0.255	-0.256

Supplementary Table 2. The variation of the bilayer splitting (Δk_z in Å^{-1}) with E and k_x. Δk_z has been determined by fitting the MDCs of the outer main bands from the ARPES intensity plots in Figs. 2**a-d** with Lorentzian functions. The values obtained from DFT for the CDW state (from Figs. 2**h-k**) and the non-CDW state (from Supplementary Fig. 8**a**) are also provided.

	ARPES				DFT				
k_x (Å^{-1}) / E (eV)	0.56	0.59	0.63	0.68	0.56	0.59	0.63	0.68	
								CDW	non-CDW
0.1	0.016	0.019	0.018	0.016	0.019	0.022	0.024	0.026	0.022
0.2	0.018	0.021	0.021	0.017	0.02	0.024	0.026	0.026	0.023
0.3	0.019	0.023	0.023	0.017	0.021	0.025	0.028	0.027	0.024
0.4	0.021	0.025	0.026	0.021	0.023	0.027	0.03	0.027	0.025
0.5	0.025	0.028	0.028	0.023	0.027	0.031	0.033	0.028	0.027

Fig. 4b). A set of $E(k_z)$ ARPES intensity plots parallel to ΓZ for different k_x in Supplemen-

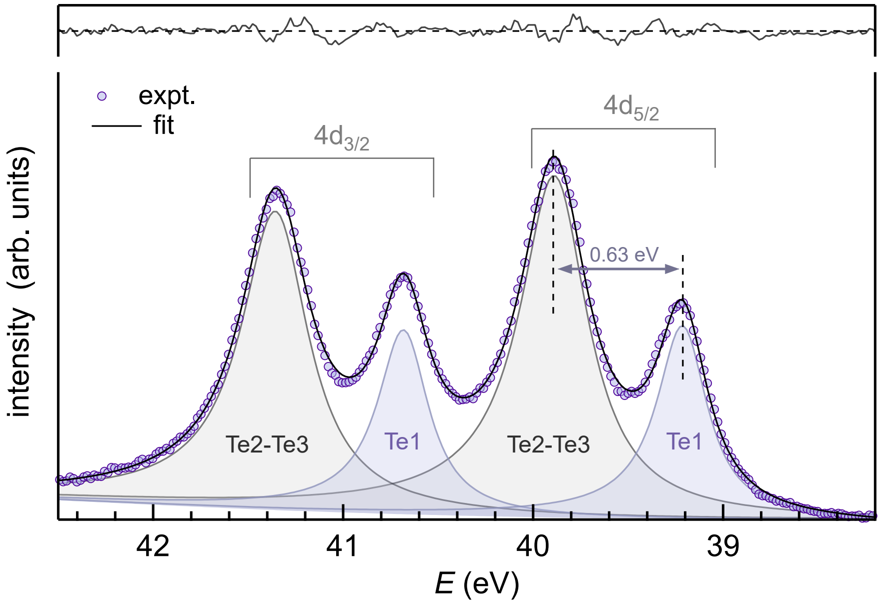

Supplementary Fig. 10. **Te 4*d* core level spectrum.** Te 4*d* core level spectrum measured using hν= 140 eV showing two spin-orbit split components corresponding to Te2-Te3 (gray shading) and Te1 (light blue shading). The least square curve fitting (solid line) has been performed using the Doniach-Sunjic lineshape as in our earlier work [11]. The residual of the fit is shown as a black curve in the top part of the figure.

tary Fig. 11a shows that the occupied band (*13*) shifts towards E_F and consequently the separation Δ decreases (Supplementary Fig. 11b). This band reaches E_F at k_x= 0.4 Å^{-1}, indicating the closure of the gap. The EBS at different k_x shown in Supplementary Figs. 11c-g provide the theoretical estimate of Δ that shows a similar trend as ARPES (Supplementary Fig. 11b). The EBS in Supplementary Fig. 11c shows that the lowest unoccupied band almost reaches E_F and provides an estimate of the CDW gap (Δ_{CDW}) to be 0.35 eV. Based on the former observation, Δ_{CDW} can be estimated from experiment to be 0.45±0.05 eV. This is in good agreement with previous ARPES study [14]. While ARPES is unable to measure Δ_{CDW} for k_x>0 since the unoccupied band moves above E_F, the estimate from DFT in Supplementary Fig. 11b shows that it remains almost unchanged up to k_x= 0.3 Å^{-1} (Supplementary Fig. 11f). In the non-CDW state, dispersing bands crossing E_F along ΓZ show the obvious absence of the CDW gap (Supplementary Fig. 12).

An $E(k_y)$ cut at $k_{||}$= 0.48 Å^{-1}(cut 1 shown in Fig. 4b) along the vertical to the k_x-k_z plane in Supplementary Fig. 11h shows the occupied band *13* (yellow arrow) that determines

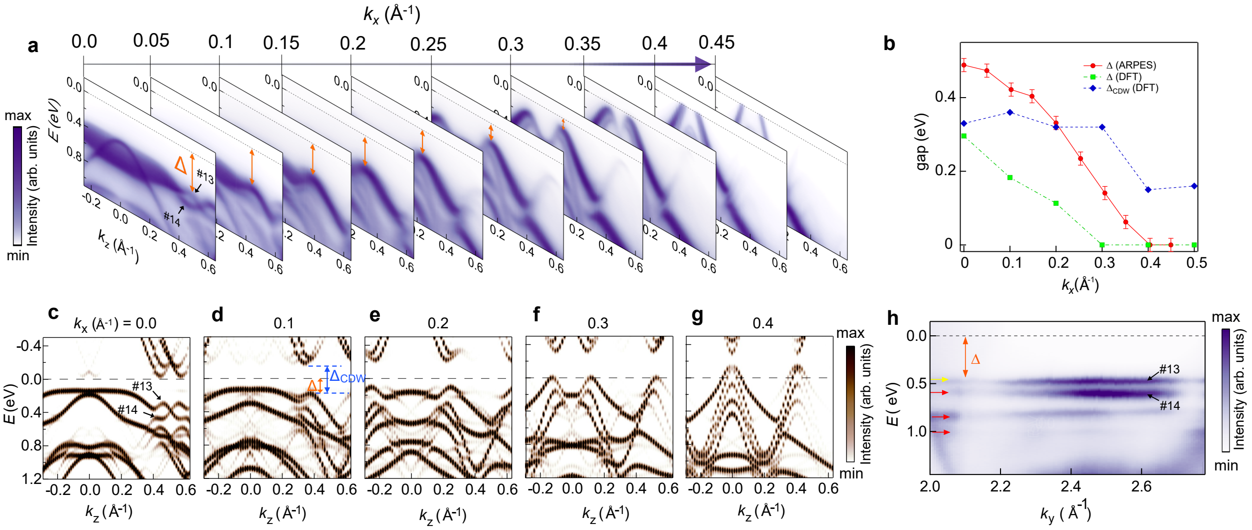

Supplementary Fig. 11. **Variation of the CDW gap. a** The $E(k_z)$ ARPES intensity plots at different k_x values. Δ indicated by the orange double arrow decreases with increasing k_x and the CDW gap closes at $k_x = 0.4\,\text{Å}^{-1}$. **b** Δ from ARPES compared with Δ_{CDW} and Δ from DFT. The error bars in Δ represent the energy resolution of ARPES. **c-g** EBS showing bands along $E(k_z)$ calculated at different k_x portraying the CDW gap variation. These panels share the same colorbar. **h** $E(k_y)$ plot measured using photon energy dependent scan (hν= 13-28 eV with 0.1 eV step) at $k_{||}$= 0.48Å^{-1}. The yellow arrow showing the occupied band (*13*) that determines Δ_{CDW} and the red arrows indicating higher E bands all remain non-dispersive along k_y showing the quasi-2D character of $LaTe_3$.

the CDW gap does not disperse. Thus the CDW gap remains unchanged along k_y. In fact, three other bands at larger E with almost no dependence on the k_y are also observed (red arrows). Absence of dispersion along k_y shows the 2D nature of $LaTe_3$.

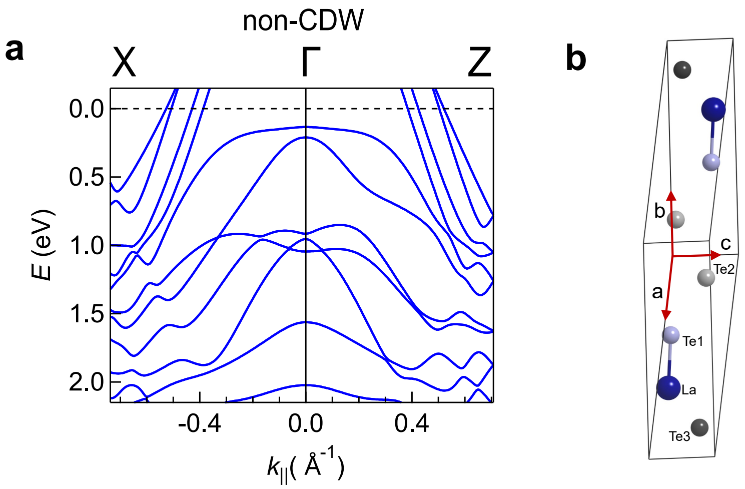

Supplementary Fig. 12. **Band structure and unit cell of $LaTe_3$ in the non-CDW state.** **a** Band dispersion along ΓX and ΓZ directions in the non-CDW state of $LaTe_3$. **b** The primitive unit cell of the non-CDW state with *Cmcm* space group is given by $a = b = 13.256$Å, $c = 4.397$Å, $\alpha = \beta = 90°$, $\gamma = 160.99°$. Note that c is 7 times less compared to c' in the CDW state shown in Fig. 1a because of the 7 fold modulation.

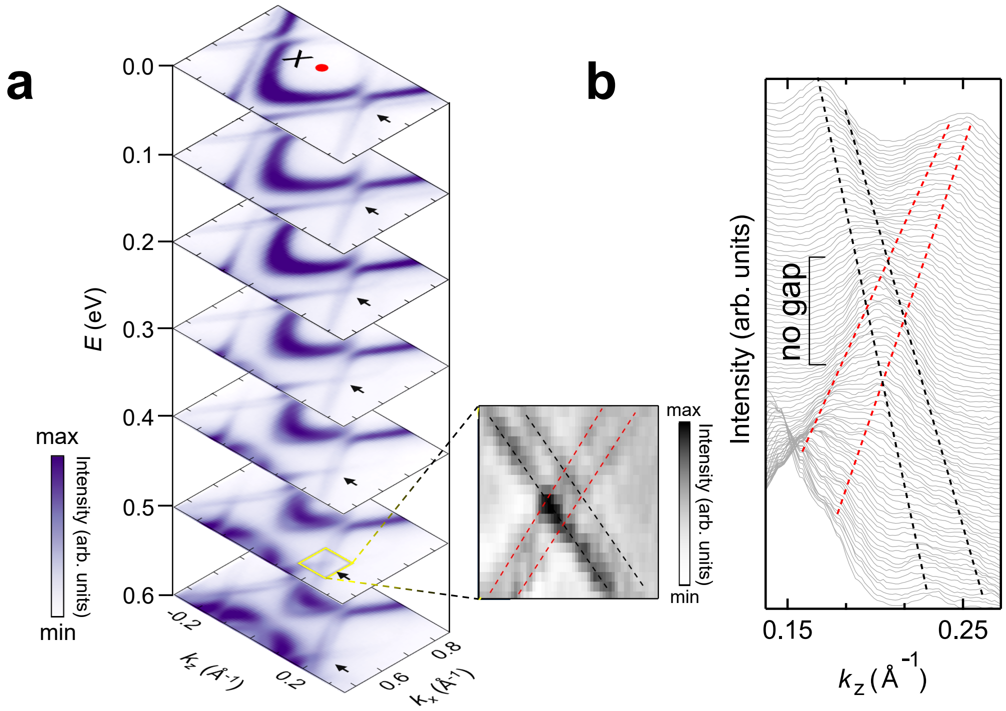

Supplementary Fig. 13. **Evidence of band crossings. a** A series of k_x-k_z isosurface plots with E varying from 0 to 0.6 eV measured around the X point. A 2D curvature image of the region marked by a yellow rectangle for $E=$ 0.5 eV highlights the crossings between the main (dashed red) and shadow (dashed black) branches. **b** A stack of MDCs taken near the crossings in Fig. 2c. The red and black dashed lines indicate the bilayer split main and shadow bands, respectively.

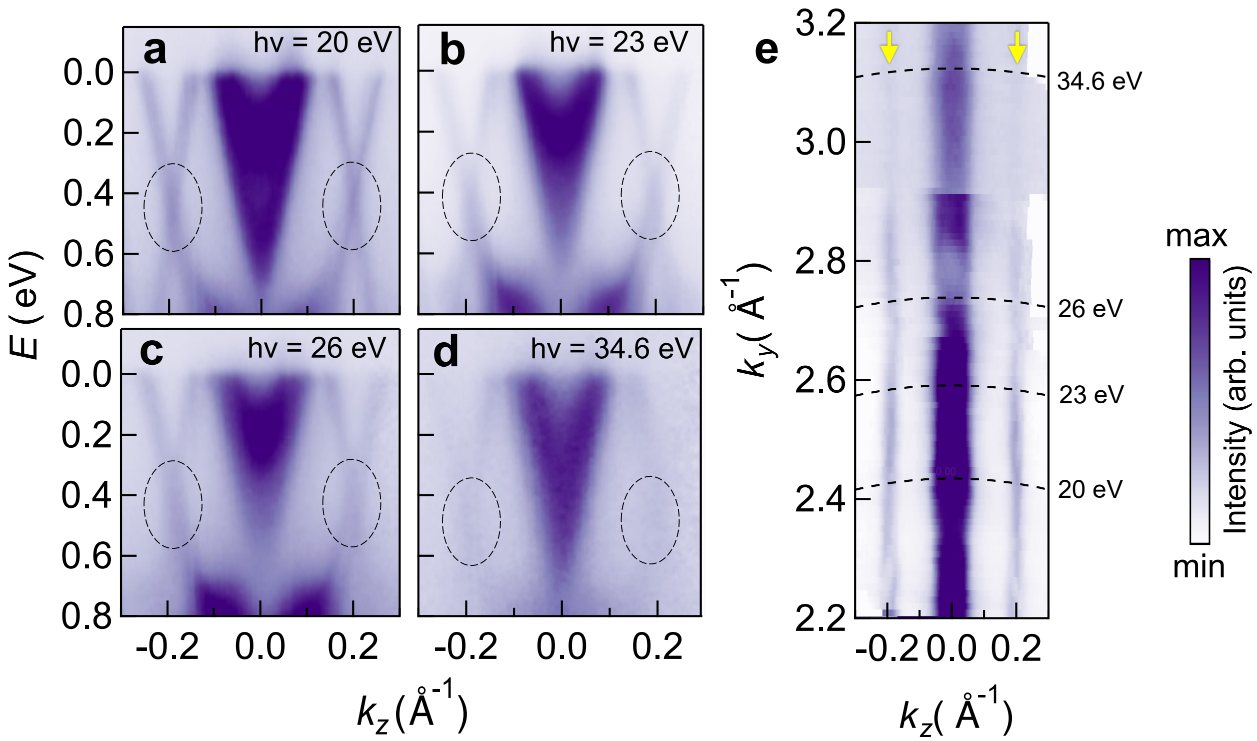

Supplementary Fig. 14. **Quasi-2D nature of the band crossings. a-d** $E(k_z)$ ARPES intensity plots at k_x=0.58 Å^{-1} with different photon energies. The band crossings are highlighted by black dashed ovals. **e** A constant energy contour at E= 0.39 eV. The effective crossings around ±0.2Å^{-1} (L and R merged together) marked by the yellow arrows do not show any dispersion as a function of hν indicating their quasi-2D nature. All the panels share the same colorbar shown on the right.

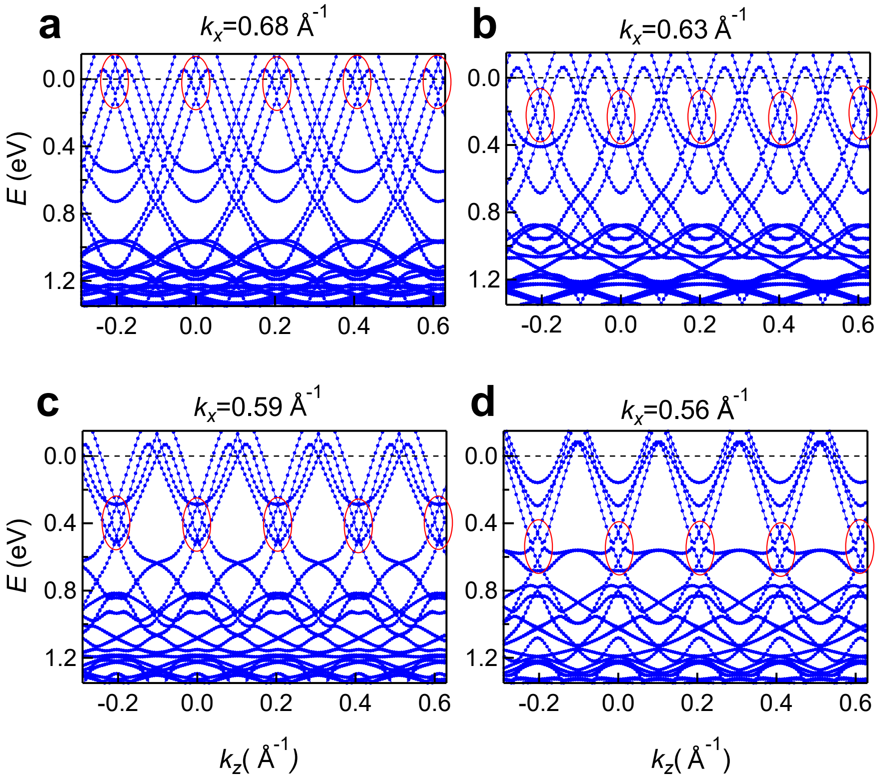

Supplementary Fig. 15. **Band structure of $LaTe_3$ in the CDW state.** The band structure of $LaTe_3$ in the CDW state for the modulated structure along k_z at k_x= **a** 0.68 Å^{-1}, **b** 0.63 Å^{-1}**c** 0.59 Å^{-1}and **d** 0.56 Å^{-1} in an extended zone scheme. The band crossing region in each BZ is highlighted by a red circle.

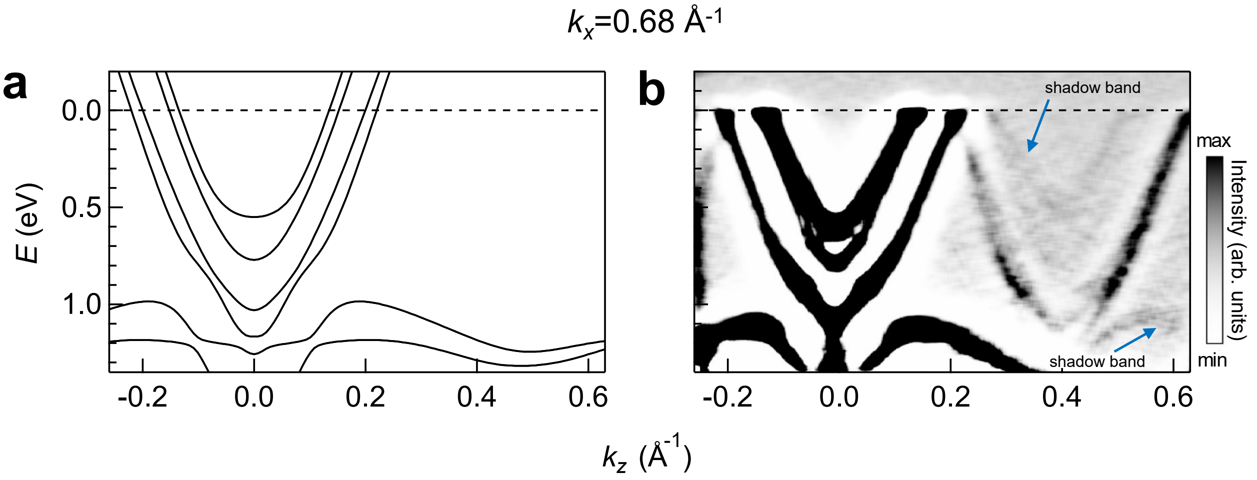

Supplementary Fig. 16. $\boldsymbol{E(k_z)}$ **bands in the non-CDW and 2D curvature plot from ARPES in the CDW state.** **a** The $E(k_z)$ bands in the non-CDW state of $LaTe_3$. **b** 2D curvature plot of Fig. 2a showing the weak shadow bands of the inner branch (marked by blue arrow).

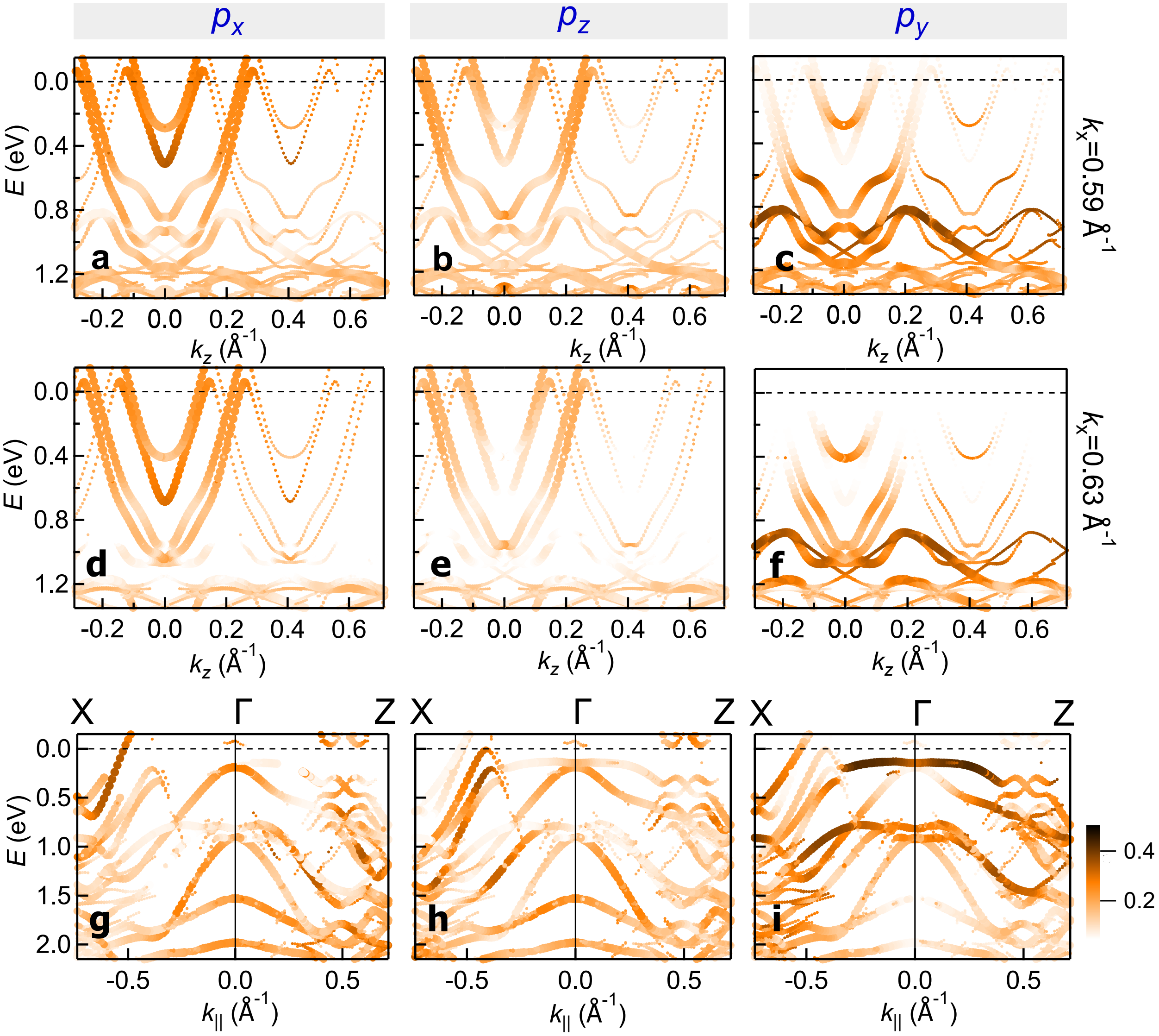

Supplementary Fig. 17. p **orbital characters of the EBS.** The p_x, p_y and p_z orbital characters of the EBS in the CDW state along k_z at **a-c** k_x= 0.59 Å^{-1}, **d-f** k_x= 0.63 Å^{-1}and **g-i** along $X\Gamma Z$. The intensity and size of the markers represent the weightage of the orbital characters and the EBS, respectively. All the panels share the same colorbar displayed to the right of panel **i**.

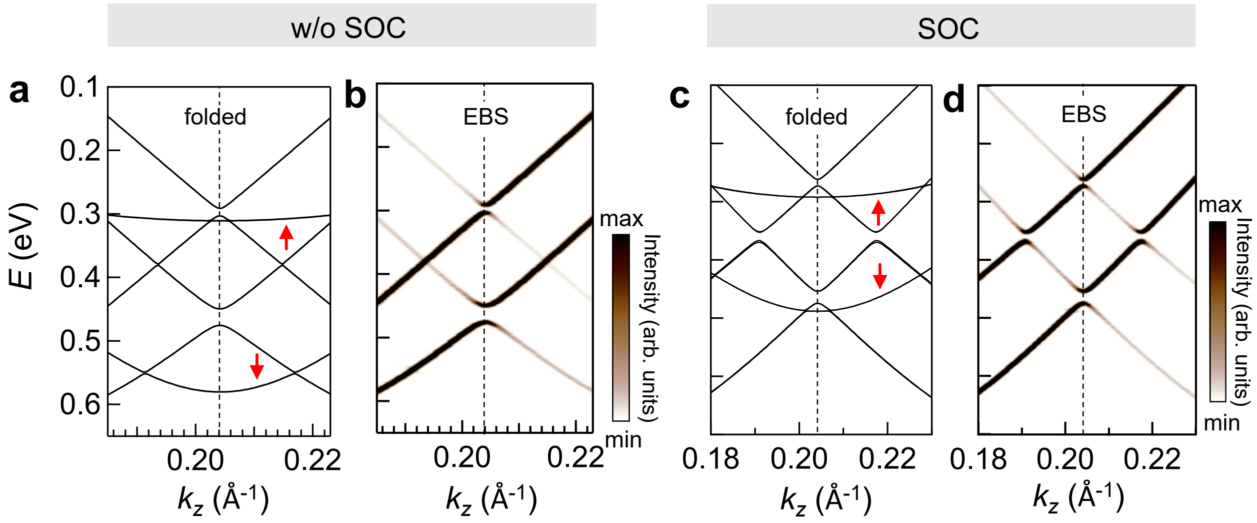

Supplementary Fig. 18. **Band structure and the EBS for the 7f structure.** The band structure of the 7f structure at k_x= 0.59 Å^{-1} w/o SOC (panel **a**) and with SOC (panel **c**), (also shown in Fig. 3**a** and **i** of the main text) is repeated here for the ease of direct comparison with the corresponding EBS shown in panels **b** and **d**, respectively. The relatively flat bands indicated by the red arrows in panels **a** and **c** are absent in the EBS due to their low spectral weights.

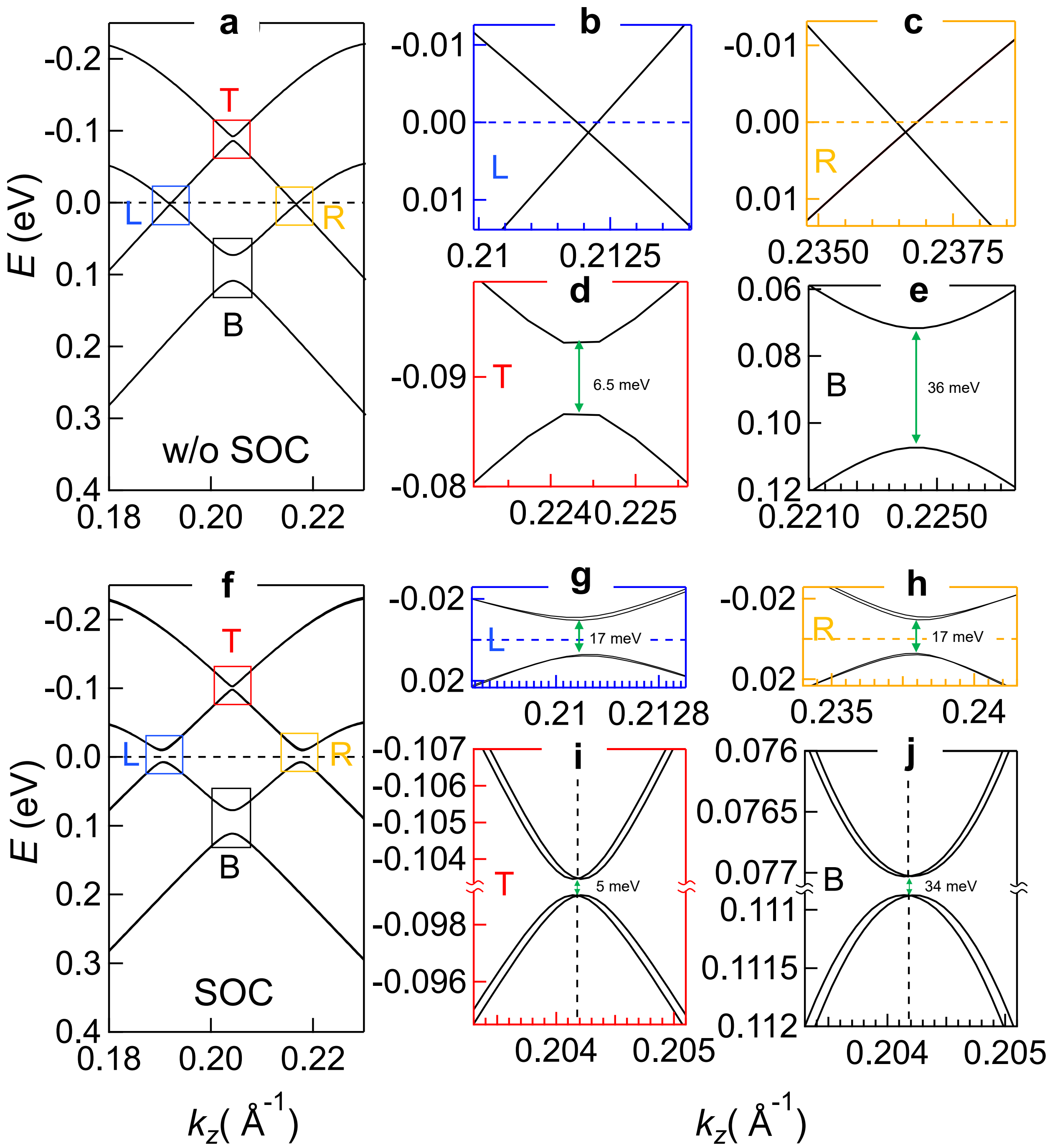

Supplementary Fig. 19. **Band structure at** k_x= **0.685 Å^{-1}. a** $E(k_z)$ bands from DFT at k_x= 0.685Å^{-1} without SOC. $E(k_z)$ bands in a small region of the k space within the colored rectangles of panel **a** around **b** L, **c** R, **d** T, and **e** B. **f-j** Same as above except that the calculations include SOC. The vertical dashed lines in **i, j** represent the k_z on the $\Gamma_2 X_2$ line.

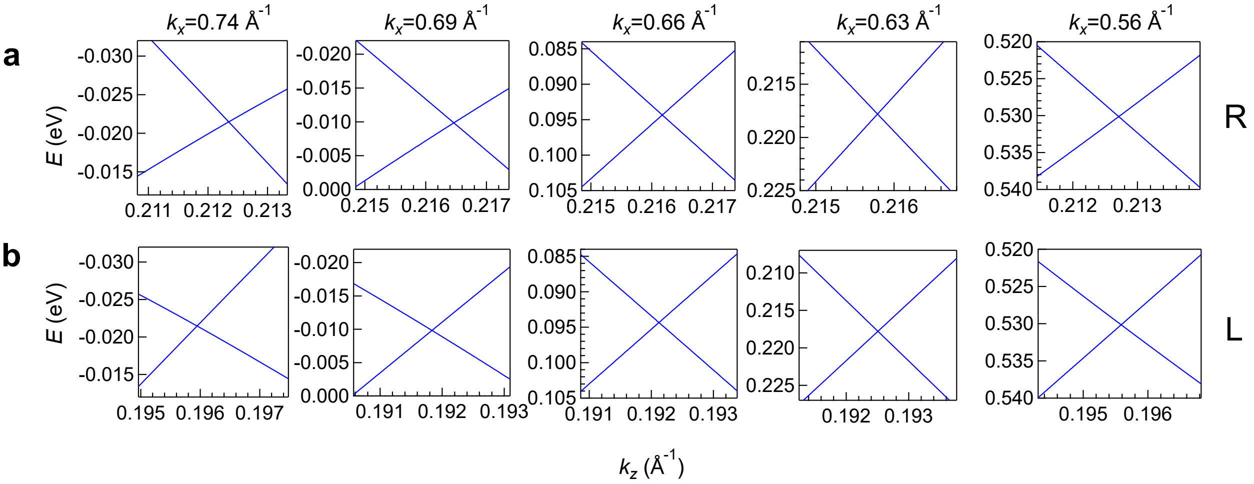

Supplementary Fig. 20. ***L* and *R* band crossings at different k_x.** A series of $E(k_z)$ bands calculated in a small region at different k_x near the **a** R and **b** L crossings without SOC.

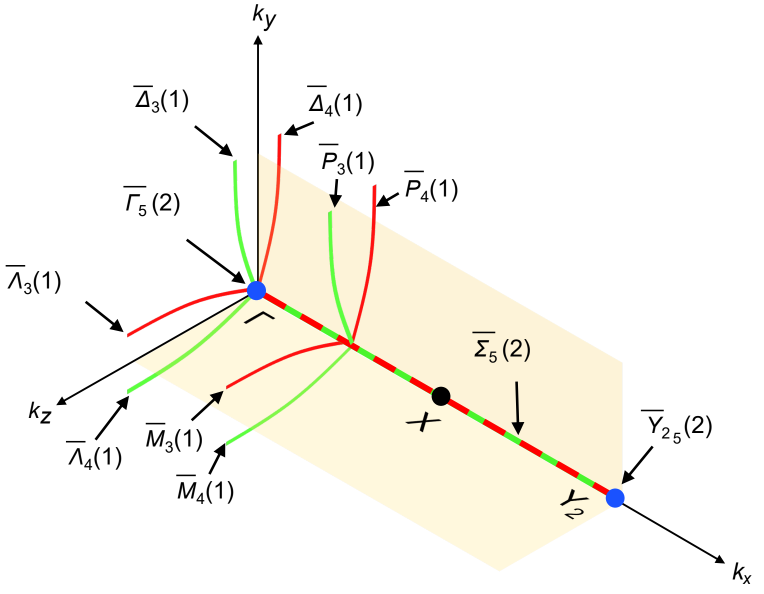

Supplementary Fig. 21. **Compatibility relations between different *k*-paths.** Compatibility relations and band connectivity for *SG* #40 double space group along different k-paths. The band splittings are shown in the energy axis. The red-green stripped line ($\overline{\Sigma}_5$) shows the Kramers nodal line along the Σ line. The TRIM points are denoted by filled blue circles.

Supplementary Table 3. Irreducible representations of SG #40 double space group [17].

Point/ line of BZ	Irrep (dim)	$\{2_{100}\|000\}$	$\{m_{010}\|00\frac{1}{2}\}$	$\{m_{001}\|00\frac{1}{2}\}$
Γ (0,0,0)	$\overline{\Gamma}_5(2)$	$\begin{pmatrix} 0 & -1 \\ 1 & 0 \end{pmatrix}$	$\begin{pmatrix} 0 & -i \\ -i & 0 \end{pmatrix}$	$\begin{pmatrix} -i & 0 \\ 0 & i \end{pmatrix}$
Σ $(u,u,0)$	$\overline{\Sigma}_5(2)$	$\begin{pmatrix} 0 & -1 \\ 1 & 0 \end{pmatrix}$	$\begin{pmatrix} 0 & -i \\ -i & 0 \end{pmatrix}$	$\begin{pmatrix} -i & 0 \\ 0 & i \end{pmatrix}$
Y_2 (0,0,0)	$\overline{Y_2}_5(2)$	$\begin{pmatrix} 0 & -1 \\ 1 & 0 \end{pmatrix}$	$\begin{pmatrix} 0 & -i \\ -i & 0 \end{pmatrix}$	$\begin{pmatrix} -i & 0 \\ 0 & i \end{pmatrix}$
M (u,u,v)	$\overline{M}_3(1)$	–	$e^{-i\pi(\frac{1}{2}-w)}$	–
	$\overline{M}_4(1)$	–	$e^{i\pi(\frac{1}{2}+w)}$	–
Λ $(0,0,u)$	$\overline{\Lambda}_3(1)$	–	$e^{-i\pi(\frac{1}{2}-w)}$	–
	$\overline{\Lambda}_4(1)$	–	$e^{i\pi(\frac{1}{2}+w)}$	–
Δ $(-u,u,0)$	$\overline{\Delta}_3(1)$	–	–	-i
	$\overline{\Delta}_4(1)$	–	–	i
P $(u,v,0)$	$\overline{P}_3(1)$	–	–	-i
	$\overline{P}_4(1)$	–	–	i

Supplementary Table 4. Compatibility relations between irreducible representations of SG #40 double space group along different k-paths.

k-path	Compatibility relations between irreps
Γ:(0,0,0)$\to\Sigma$:(u,u,0)	$\overline{\Gamma}_5(2)\to\overline{\Sigma}_5(2)$
Σ:(u,0,0)$\to Y$:(1,0,0)	$\overline{\Sigma}_5(2)\to\overline{Y}_5(2)$
Γ:(0,0,0)$\to\Lambda$:(0,0,u)	$\overline{\Gamma}_5(2)\to\overline{\Lambda}_3(1)\oplus\overline{\Lambda}_4(1)$
Γ:(0,0,0)$\to\Delta$:($-u$,u,0)	$\overline{\Gamma}_5(2)\to\overline{\Delta}_3(1)\oplus\overline{\Delta}_4(1)$
Σ:(u,0,0)$\to M$:(u,u,v)	$\overline{\Sigma}_5(2)\to\overline{M}_3(1)\oplus\overline{M}_4(1)$
Σ:(u,0,0)$\to P$:(u,v,0)	$\overline{\Sigma}_5(2)\to\overline{P}_3(1)\oplus\overline{P}_4(1)$

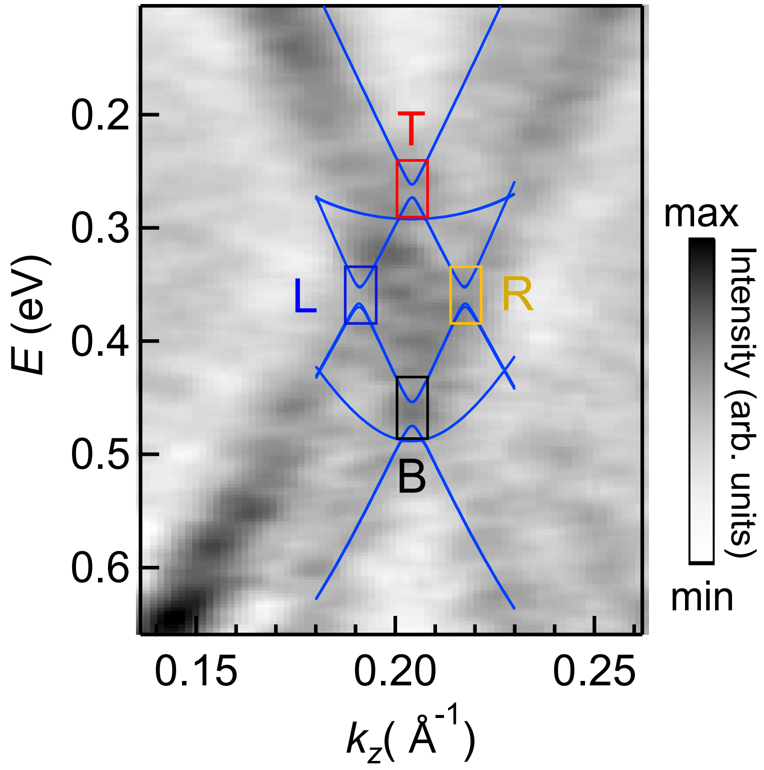

Supplementary Fig. 22. **Superposition of DFT bands on ARPES.** $E(k_z)$ band dispersion (blue curves) calculated with SOC in Fig. 3**i** is superimposed on the ARPES intensity plot in Fig. 2**f**, both at k_x= 0.59 Å^{-1}. The regions zoomed in Figs. 3**l,m** are shown within the red and black rectangles, respectively.

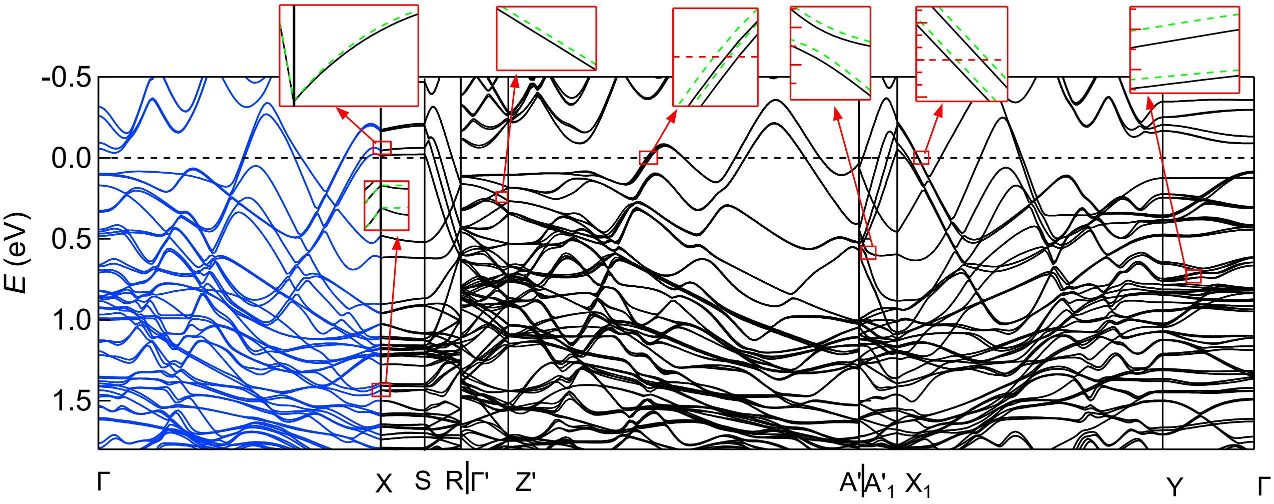

Supplementary Fig. 23. **Band structure of $LaTe_3$ along the KNL and other high symmetry directions.** Band dispersion calculated with SOC, the high symmetry points of the CDW BZ are indicated at the bottom. The two fold degenerate bands are denoted by blue curves along the KNL (ΓX). Zoomed regions from the red rectangles that are arbitrarily chosen show the band splittings in all the other directions.

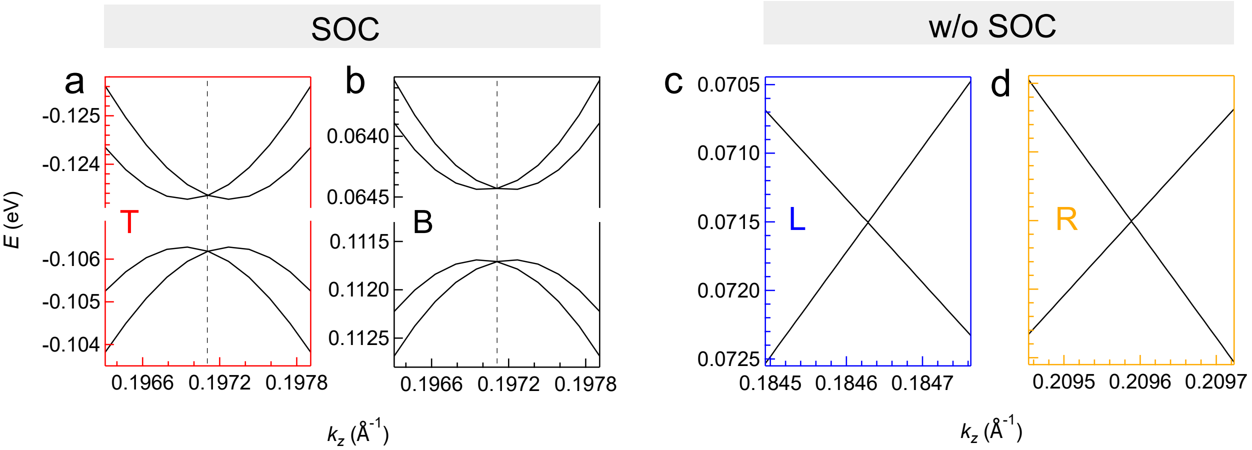

Supplementary Fig. 24. **Band crossings for 29f $LaTe_3$ at k_x= 0.685 Å^{-1}.** Band dispersion with SOC around **a** T and **b** B. The crossings w/o SOC at **c** L and **d** R.

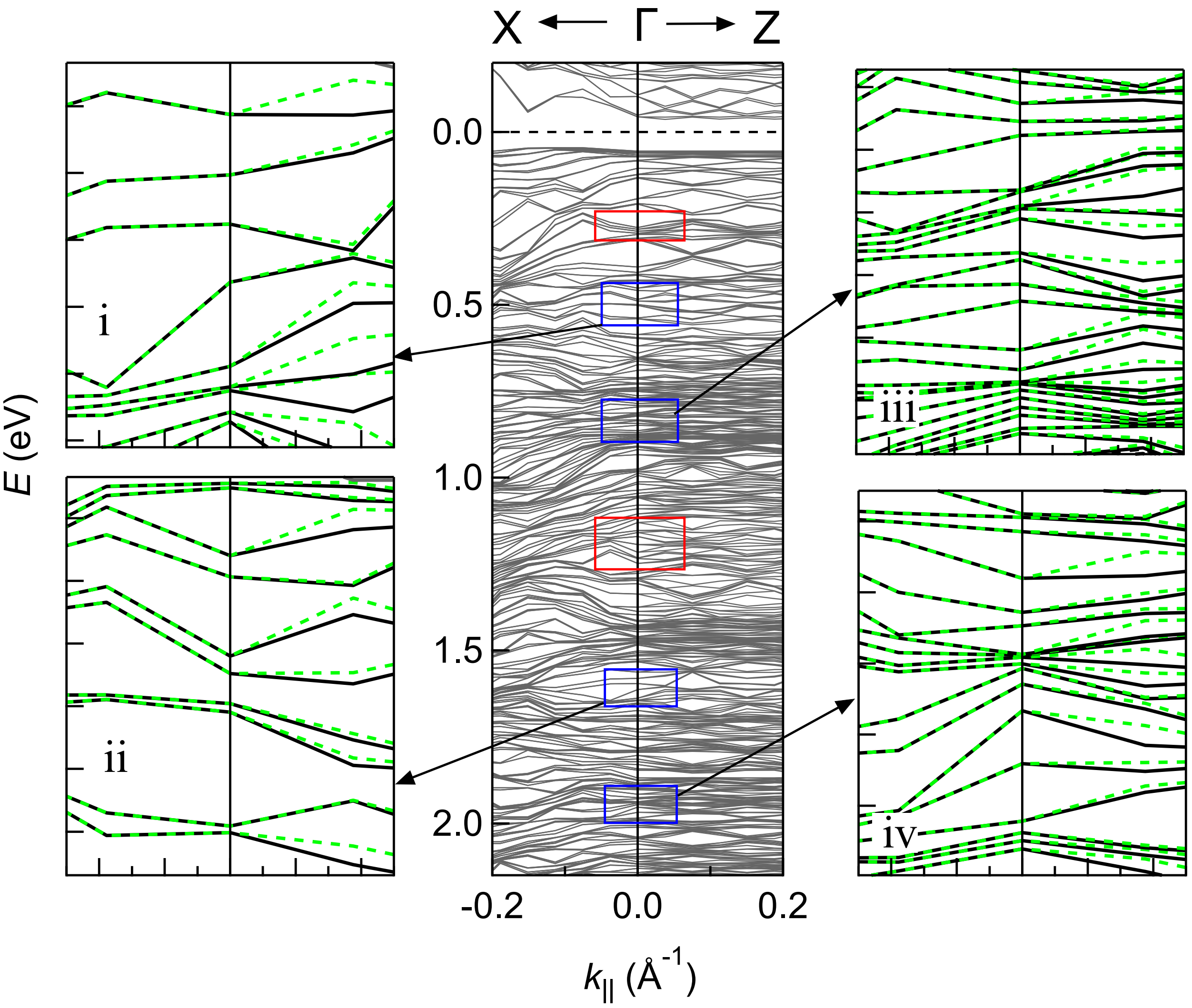

Supplementary Fig. 25. **Band dispersion of 29f** $\mathbf{LaTe_3}$ **along and perpendicular to the KNL.** The band dispersion from Γ towards the ΓX and ΓZ directions. Zoomed blue rectangles (*i*-*iv*) show the two fold degeneracy (dashed-green and black) of the bands along the KNL (ΓX), whereas in contrast the degeneracy is lifted along ΓZ. The red rectangle regions are shown in the main text in Fig. 5**e,f**.

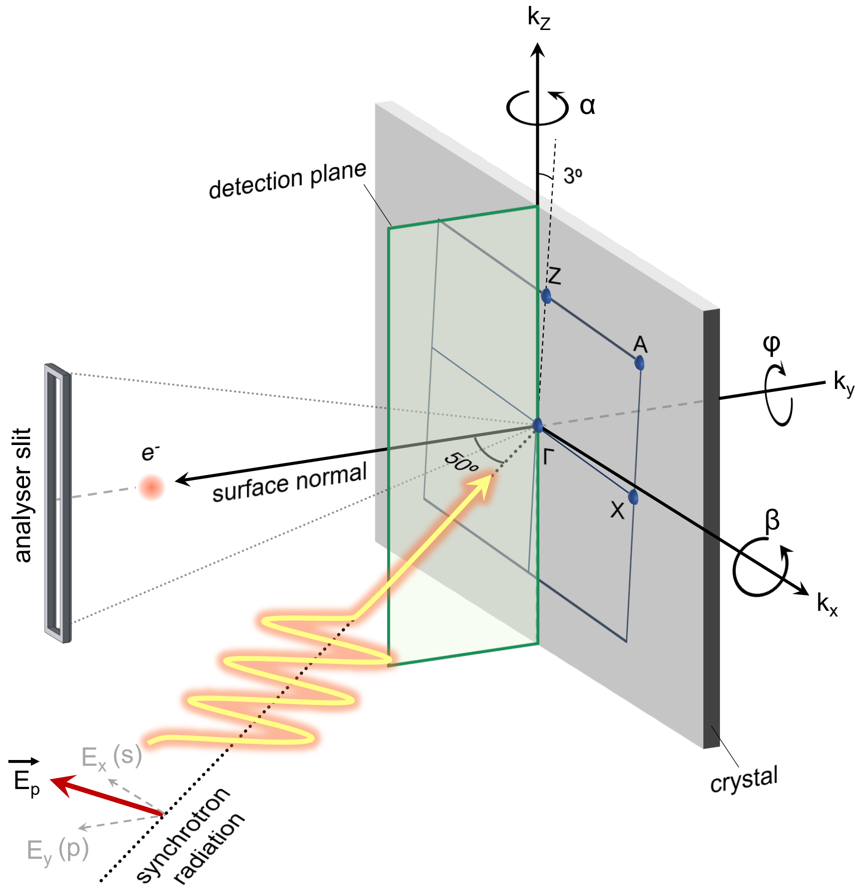

Supplementary Fig. 26. **Experimental geometry of the ARPES measurements.** The linearly polarized photon beam is incident in the horizontal plane with an angle of 50° with the surface normal oriented along the analyzer axis. The analyzer slit was oriented vertically, and the vertical detection plane is shown by green color. The E_y (E_x) component of the photon polarization vector ($\mathbf{E}_p$) is parallel (perpendicular) to the detection plane providing p (s) polarized light. The blue square depicts the BZ of the non-CDW state. The crystal was azimuthally (ϕ) rotated to align the analyzer slit towards the ΓZ direction. The Fermi surface was mapped by varying the polar angle (α).

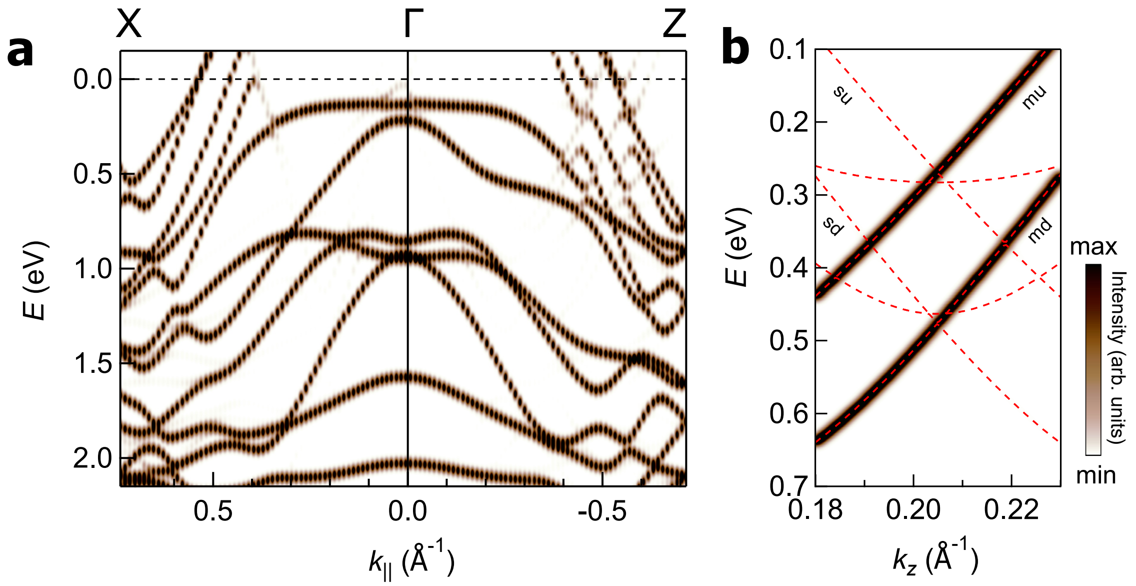

Supplementary Fig. 27. **EBS and band structure of the 7f structure with geometry optimization.** **a** The EBS showing absence of the CDW gap along ΓZ. **b** The $E(k_z)$ band structure (red dashed curves) calculated at k_x= 0.59 Å^{-1} with SOC shows both the main and shadow bands. However, the EBS only displays the main bands (*mu* and *md*) because the shadow bands have much lower spectral weight. Both panels share the same colorbar shown on the right.

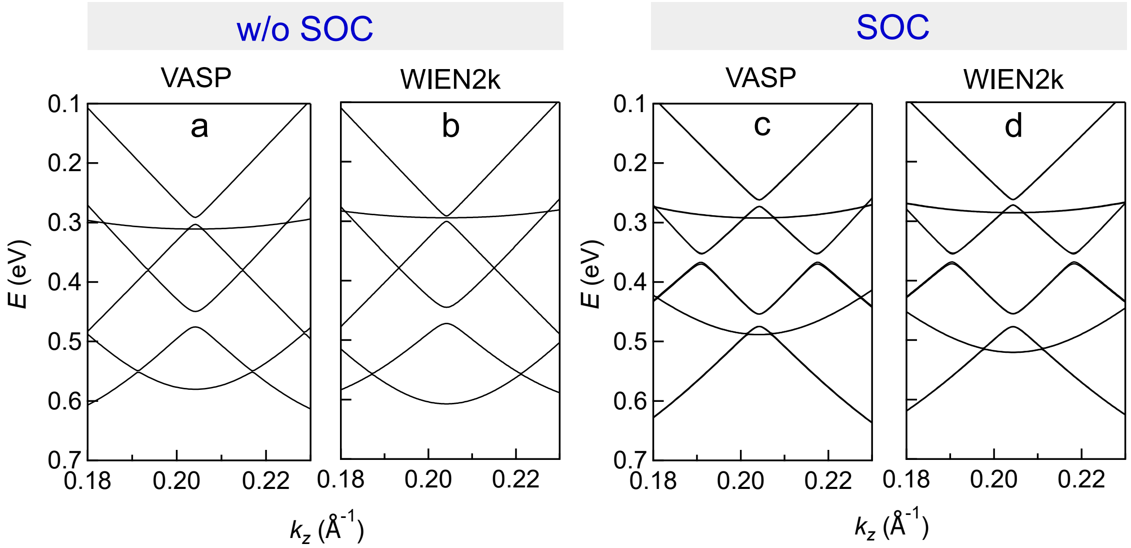

Supplementary Fig. 28. **Band structures obtained from VASP and WIEN2k.** Comparison of the bands along k_z at k_x= 0.59 Å^{-1} in the CDW state calculated using **a,c** VASP within the projector augmented method and **b,d** WIEN2k that is a full-potential linear augmented plane wave method based on all-electron calculations. See the **Methods** section for the details.

Supplementary References

[1] Malliakas, C. D. and Kanatzidis, M. G. Divergence in the behavior of the charge density wave in $RETe_3$ (RE = rare-earth element) with temperature and RE element. *Journal of the American Chemical Society* **128**, 12612 (2006).

[2] Fei, Z. et al. Ferroelectric switching of a two-dimensional metal. *Nature* **560**, 336 (2018).

[3] Kopaczek, J. et al. Experimental and theoretical studies of the surface oxidation process of rare-earth tritellurides. *Advanced Electronic Materials* **9**, 2201129 (2023).

[4] Lavagnini, M. et al. Evidence for coupling between charge density waves and phonons in two-dimensional rare-earth tritellurides. *Phys. Rev. B* **78**, 201101 (2008).

[5] Zhang, K. et al. Raman signatures of inversion symmetry breaking and structural phase transition in type-II weyl semimetal $MoTe_2$. *Nat. Commun.* **7**, 13552 (2016).

[6] Setyawan, W. and Curtarolo, S. High-throughput electronic band structure calculations: Challenges and tools. *Computational Materials Science* **49**, 299 (2010).

[7] Komoda, H. et al. High-resolution angle-resolved photoemission study of incommensurate charge-density-wave compound $CeTe_3$. *Phys. Rev. B* **70**, 195101 (2004).

[8] Laverock, J. et al. Fermi surface nesting and charge-density wave formation in rare-earth tritellurides. *Phys. Rev. B* **71**, 085114 (2005).

[9] Garcia, D. R. et al. Revealing charge density wave formation in the $LaTe_2$ system by angle resolved photoemission spectroscopy. *Phys. Rev. Lett.* **98**, 166403 (2007).

[10] Shin, K. Y., Brouet, V., Ru, N., Shen, Z. X. and Fisher, I. R. Electronic structure and charge-density wave formation in $LaTe_{1.95}$ and $CeTe_{2.00}$. *Phys. Rev. B* **72**, 085132 (2005).

[11] Sadhukhan, P. et al. Electronic structure of Au-Sn compounds grown on Au(111). *Phys. Rev. B* **100**, 235404 (2019).

[12] Bader, R. F. Atoms in molecules,. *Accounts of Chemical Research* **18**, 9 (1985).

[13] Bhattacharya, J. and Chakrabarti, A. Electronic and transport properties of heusler alloy based magnetic tunneling junctions: A first principles study. *Computational Materials Science* **216**, 111852 (2023).

[14] Brouet, V. et al. Angle-resolved photoemission study of the evolution of band structure and charge density wave properties in RTe_3 (R=Y, La, Ce, Sm, Gd, Tb, and Dy). *Phys. Rev. B* **77**, 235104 (2008).

[15] Sarkar, S. et al. X-ray photoelectron spectroscopy study of a layered tri-chalcogenide system $LaTe_3$. *AIP Conference Proceedings*, **2220**, 100005 (2020).

[16] Brouet, V. et al. Fermi surface reconstruction in the CDW state of $CeTe_3$ observed by photoemission. *Phys. Rev. Lett.* **93**, 126405 (2004).

[17] Elcoro, L. et al. Double crystallographic groups and their representations on the Bilbao crystallographic server. *Journal of Applied Crystallography* **50**, 1457 (2017).
