# Supplementary material for: Charge density wave induced nodal lines in LaTe3
Source: Nat Commun. 2023 Jun 19;14:3628. doi: 10.1038/s41467-023-39271-1 (PMC10279677; doi:10.1038/s41467-023-39271-1)
Supplement: Supplementary file 3 — Description of Additional Supplementary files [file 41467_2023_39271_MOESM3_ESM.docx]

Inventory of Supporting Information

1. Supplementary Information file named `LaTe3_SM_R3_NatComm` comprising of Supplementary Figures 1-28, Supplementary Tables 1-4, and Supplementary Notes 1-5.
2. Supplementary Data 1: Structure (.cif) file named `29fLaTe3` for 29 fold LaTe_3_
